# Future increase in elderly heat-related mortality of a rapidly growing Asian megacity

**DOI:** 10.1038/s41598-020-66288-z

**Published:** 2020-06-09

**Authors:** Alvin Christopher G. Varquez, Nisrina S. Darmanto, Yasushi Honda, Tomohiko Ihara, Manabu Kanda

**Affiliations:** 1 Department of Transdisciplinary Science and Engineering, Tokyo Institute of Technology, Tokyo, Japan; 2 Faculty of Health and Sport Sciences, University of Tsukuba, Tsukuba, Japan; 3 Department of Environment Systems, University of Tokyo, Tokyo, Japan

**Keywords:** Climate change, Climate-change impacts, Climate-change mitigation, Risk factors

## Abstract

Urban dwellers are at risk of heat-related mortality in the onset of climate change. In this study, future changes in heat-related mortality of elderly citizens were estimated while considering the combined effects of spatially-varying megacity’s population growth, urbanization, and climate change. The target area is the Jakarta metropolitan area of Indonesia, a rapidly developing tropical country. 1.2 × 1.2 km^2^ daily maximum temperatures were acquired from weather model outputs for the August months from 2006 to 2015 (present 2010s) and 2046 to 2055 (future 2050s considering pseudo-global warming of RCP2.6 and RCP8.5). The weather model considers population-induced spatial changes in urban morphology and anthropogenic heating distribution. Present and future heat-related mortality was mapped out based on the simulated daily maximum temperatures. The August total number of heat-related elderly deaths in Jakarta will drastically increase by 12~15 times in the 2050s compared to 2010s because of population aging and rising daytime temperatures under “compact city” and “business-as-usual” scenarios. Meanwhile, mitigating climate change (RCP 2.6) could reduce the August elderly mortality count by up to 17.34%. The downwind areas of the densest city core and the coastal areas of Jakarta should be avoided by elderly citizens during the daytime.

## Introduction

Numerous studies^1–5^ have confirmed with sufficient evidence that heat-related mortality or morbidity rates is a function of ambient temperature. While multiple factors affect changes in ambient temperature, heatwave and urban heat island (UHI) phenomena^6^, in particular, are considered detrimental to mortality, especially among elderly^7^ and low-income groups^8–10^ (arguably regional dependent^11^, within cities. Heatwave is a prolonged period of extremely hot weather, while UHI is characterized by excess warming of urban areas relative to its surrounding rural areas. Both phenomena are interrelated^12^ and are largely affected by time-varying background climate^13,14^ and the level of urbanization^15^ of a city.

Effects of climate change to heat-related mortality or morbidity rates have been widely investigated^16–20^. An earlier study by Hales *et al*.^1^ suggested that low adaptation-mitigation strategic implementations may lead to 0.1~0.6% heat-related excess deaths in the 2050s with Asian countries being the most vulnerable. Under certain temperatures, heat-related mortality relative risk (*RR*), defined by the ratio of incidence of dying with or without exposure to extreme temperatures, was found to be a function of temperature. Above a certain optimum temperature (OT), *RR* increases proportionally^21–23^.

Despite the increasing information of *RR* globally^23^, future heat-related mortality projections attributed to heat stress remain coarsely investigated (e.g. bulk estimates with coarse spatial resolutions of ~30-km in France^24^ and are few in quantity^25^. At this coarse resolution, it makes it difficult to provide specific adaptation and mitigation measures up to a neighborhood scale. Also, mapping out heat-related mortality as a function of temperature distribution is challenging because most existing weather models lack consideration of urban sprawling or population growth distributions at neighborhood scales. Precise spatial mapping of heat-related health risks considering both urbanization and climate change are needed.

A climate change study by Darmanto *et al*.^26^ was recently conducted for the Jakarta metropolitan area while investigating the combined effects of urbanization and climate change to 2050s climate. Urbanization was represented by spatial building morphological and anthropogenic emission changes, directly related to population changes. In their work, they considered present (i.e. PRESENT) and two future climate change scenarios. Future scenarios were defined by feasible plausible combinations of shared-socioeconomic pathways (for urbanization) and representative concentration pathways (for climate). One 2050s future scenario (RCP26CC) was characterized by a condition of best adaptation and mitigation strategy implementation, low emissions, high-energy efficiency, high resilience, with controlled urban growth. Another future scenario (RCP85BaU) was characterized by low adaptation and mitigation strategy implementation with unrestricted future urbanization.

In this work, we aim to contribute to the lack of kilometer grid-scale investigations on present and future heat-related mortality. To achieve this and to overcome the challenge of fine-scale future projections of heat-related mortality, we investigated and mapped present (2010s) and future (2050s) heat-related mortality of elderly citizens in Jakarta while utilizing August temperature outputs of Darmanto *et al*.^24^ at almost 1-km grid scales. August is in the middle of the dry season^27^ in Jakarta, the capital of Indonesia. Jakarta, is one of the top 30 largest cities with a total population exceeding 10 million in 2015 and is projected to continually rise^28^. Indonesia is an emerging middle-income country expecting multiple climate-change vulnerabilities^29^. Although awareness is common for directly perceivable environmental issues such as flooding, land subsidence, and air pollution, majority of the capital’s population remains potentially at risk to heatwave and urban warming due to lack in financial capacity to purchase air-conditioning^30^ (see methods and supplementary information for the detailed explanation on the cases and the analyses process).

## Results

### Heat-related elderly maps of Jakarta in 2010s vs 2050s

**Figure 1:**
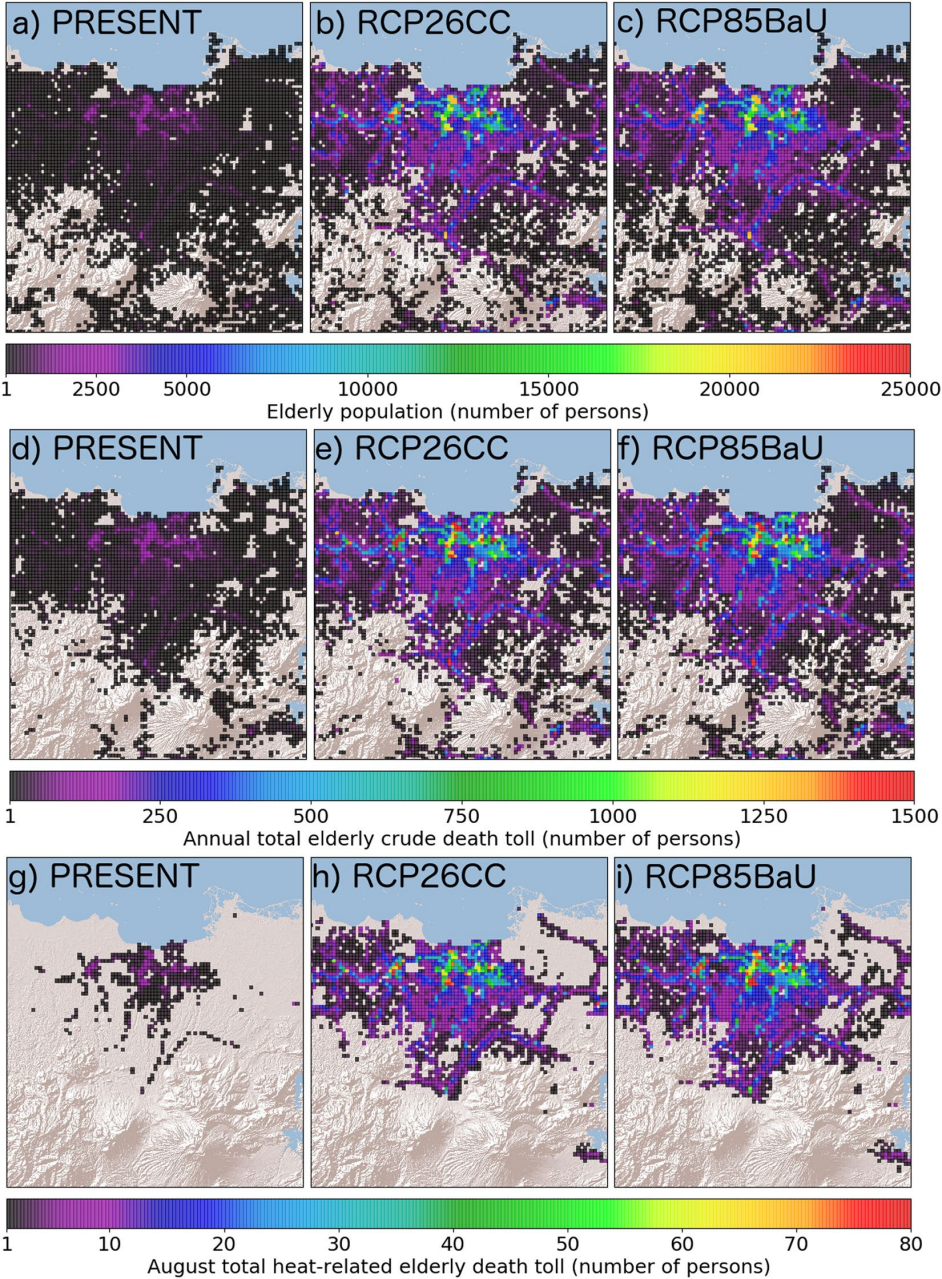
(**a–c**) correspond to estimated elderly population per grid for PRESENT, RCP26CC, RCP85BaU, respectively. (**d**,**e**), f represent annual total elderly mortality count per grid for PRESENT, RCP26CC, RCP86BaU, respectively. (**g–i**) show August total heat-related elderly mortality count per grid for PRESENT, RCP26CC, RCP86BaU, respectively.

The present-day elderly population and the projected increase (= *r*65*pop, see methods) in Jakarta, and their corresponding heat-related death estimates are mapped out as shown in Fig. 1. The percentage of the elderly population (persons whose ages are above 65 years) in Jakarta to its total population is expected to increase from 3.33% (PRESENT) to 22.03% (future cases) depicting an aging society (see methods). The estimated total population of elderly people in Jakarta are 751127, 7348107, and 7739856 for PRESENT, RCP26CC, and RCP85BaU, respectively. Incidentally, the estimated total crude deaths of elderly citizens are 53251, 520943, 548717 for PRESENT, RCP26CC, and RCP85BaU. The total August mean estimated heat-related mortality counts of elderly citizens are 1776, 22198, and 26856, for PRESENT, RCP26CC, and RCP85BaU, respectively.

Comparing the absolute number of heat-related mortality between the PRESENT and future cases (RCP26CC and RCP85BaU) reveals a 2050s projected increase by 12 to 15 times of 2010s heat-related elderly mortality count. This increase is closely proportional to the increase in the estimated elderly population between the 2010s and 2050s which is by a factor of 9 to 10. For every 1.2 × 1.2 sq. km. district in Jakarta with populations greater than or equal to 1000, a mean of 0.75 ± 1.07, 8.27 ± 11.20, 9.28 ± 12.36 heat-related elderly mortality counts for PRESENT, RCP26CC, and RCP85BaU was estimated in August with larger counts at more populated areas. These significant differences suggest that the large population increase of vulnerable elderly citizens plays a dominant role in the drastic increase of heat-related mortality counts.

Interestingly, the proportion of August total heat-related mortality counts of elderly citizens to the annual total elderly population crude death varies for each case with RCP85BaU having the highest proportion of 4.89%, followed by RCP26CC of 4.26%, and the PRESENT of 3.33%. This increasing proportion from the 2010s to 2050s is caused by the increasing hazard due to rising daily maximum temperatures under the assumed climate change pathways (see the daytime maximum temperature in methods and supplementary information). Comparing the proportions of the future cases, RCP26CC is lower by 17.34% compared to RCP85BaU. As mentioned earlier, the future scenario represented by RCP26CC is a pathway by which the government strongly imposes aggressive plans for mitigation and adaptation to climate change. If the Indonesian government imposes aggressive mitigation and adaptation strategies as defined by the RCP 2.6 climate scenario (e.g. compact city), expected casualties in the future reduce by 17.34% compared to the worst-case (RCP 8.5) scenario.

### Mortality impact of future warming in Jakarta to present-day elderly population under extreme (RCP 8.5) and controlled (RCP 2.6) future climate scenarios

**Figure 2:**
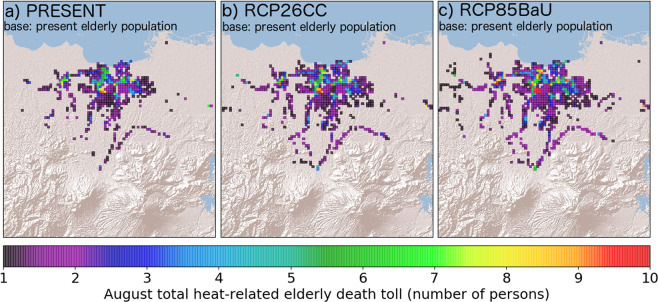
Estimated heat-related elderly mortality count when exposing the present-state population of elderly citizens to maximum temperatures simulated in (**a**) PRESENT, (**b**) RCP26CC, and (**c**) RCP85BaU cases for August months. Uncolored grids mean zero heat-related elderly deaths.

Exacerbated by the increase in August daily maximum temperature (0.51 ± 0.36 °C and 1.16 ± 0.38 °C for RCP26CC and RCP85BaU, respectively) at populated areas, a drastic increase in estimated heat-related elderly mortality count in 2050s is driven by the projected increase in the vulnerable elderly population mentioned in Sect. 2.1. To investigate the effect of climate change on the mortality count without the influence of population aging, heat-related mortality of the current elderly population was estimated while considering each cases’ *T*_*max*_ distribution. The results are summarized in Fig. 2. The grids with heat-related mortality counts of elderly citizens widen under varying future scenarios with RCP85BaU revealing an increase in heat-related mortality at sprawled areas. In the PRESENT case, casualties are mostly concentrated southwest of the city center. the August total heat-related elderly mortality count was 1776 (3.33% of estimated total elderly crude deaths of PRESENT), 2213 (4.16%), and 2564 (4.82%) for PRESENT, RCP26CC, and RCP85BaU, respectively, which is almost 10 times lower than when future elderly population is considered. An increase in the total mortality count by a factor of 1.25 and 1.44 was found when the current population was exposed to RCP26CC and RCP85BaU climate, respectively. The increase in mortality count under RCP26CC suggests that despite strict measures of the government to mitigate and adapt to climate change, heat-related risks are still to be expected for elderly citizens.

### Future projection of heat-related risks for climate change adaptation

**Figure 3:**
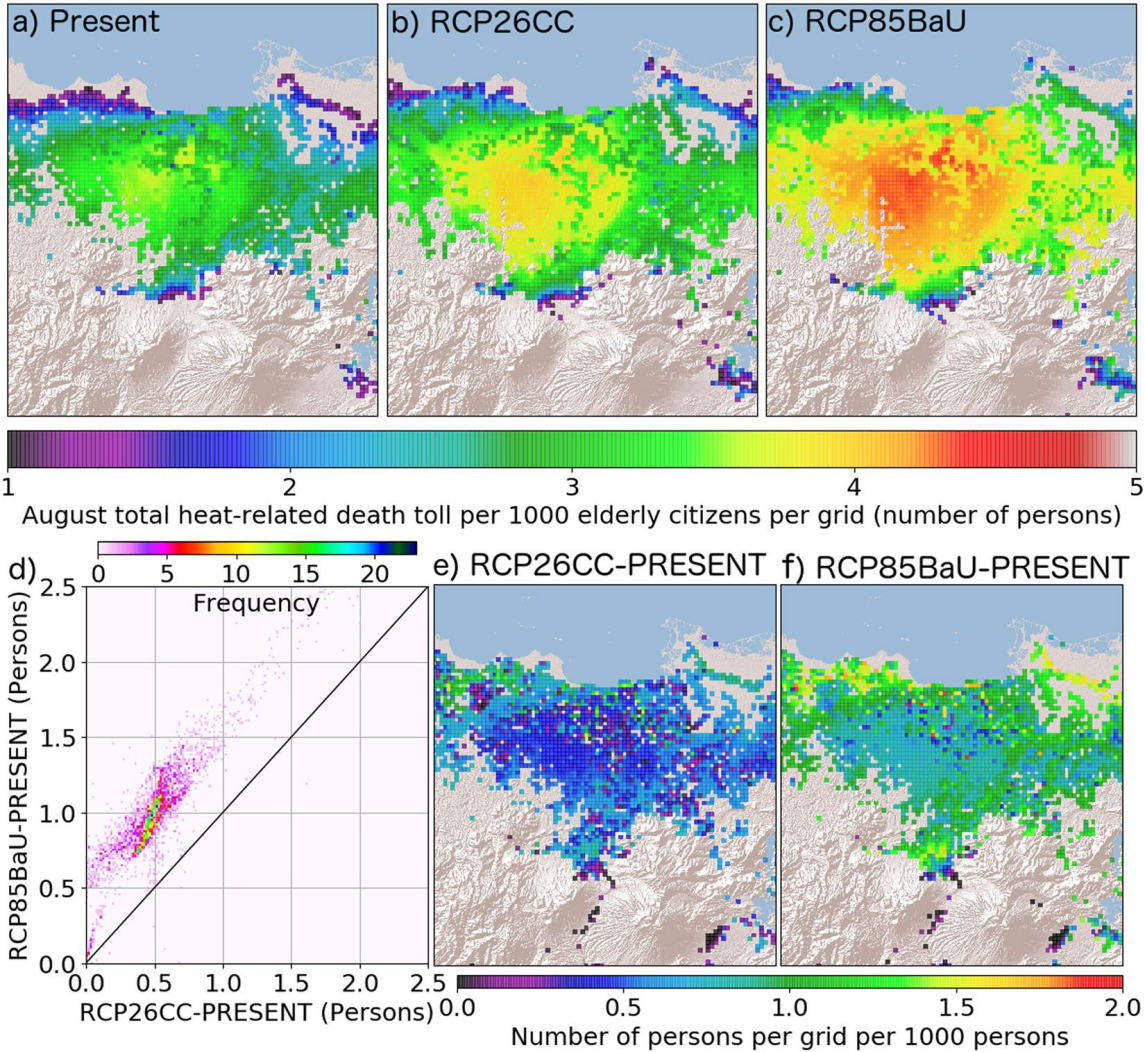
August total heat-related mortality count per 1000 elderly citizens per grid. (**a–c**) correspond to PRESENT, RCP26CC, and RCP85BaU cases. A 2-dimensional histogram (**d**) shows the changes in the death toll per 1000 elderly citizens per grid from 2010s to 2050s under RCP26CC (**e**) and RCP85BaU (**f**) scenarios.

**Figure 4:**
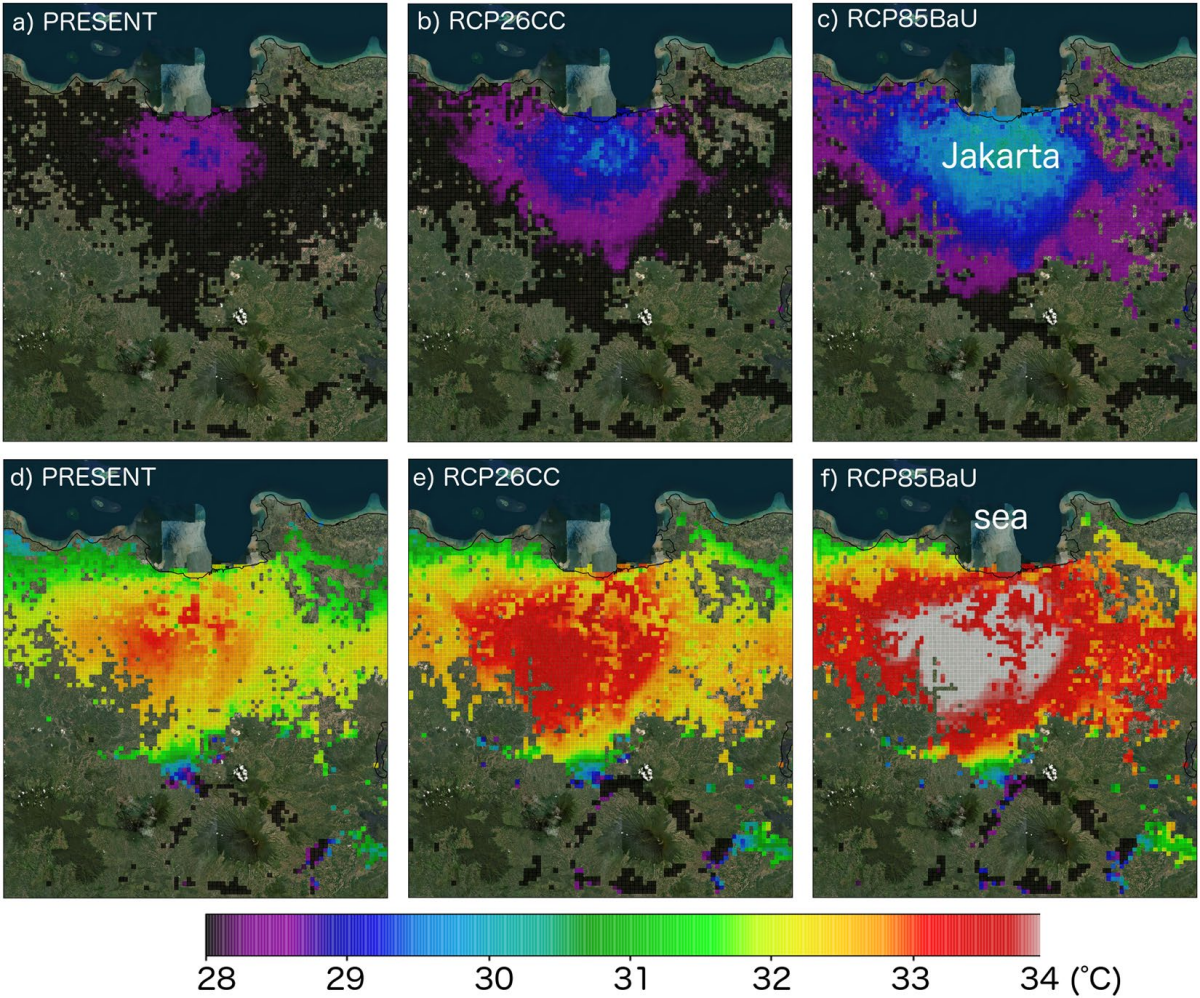
August-average of daily mean (**a–c**) and daily (**d–f**) maximum bias-corrected near-surface temperature at urban grids of Jakarta metropolitan area for PRESENT (**a**,**d**), RCP26CC (**b**,**e**), and RCP85BaU (**c**,**f**) cases.

To locate areas that can potentially be risky in terms of future heat-related mortality and morbidity, August total heat-related mortality count of elderly citizens while assuming 1000 elderly citizens for all analyzed populated grids was estimated. Figure 3 shows the analyses results and the differences for each case. On average, the August total heat-related mortality count of elderly citizens per 1000 elderly citizens are 2.44 ± 0.93, 2.79 ± 1.08, 3.25 ± 1.14 persons for PRESENT, RCP26CC, and RCP85BaU, respectively. Vaguely noticeable when utilizing actual population distribution (Fig. 1), potentially risky areas, as indicated by the map of mortality count per 1000 elderly citizens (Fig. 3a–c), are located at the southwestern areas of Jakarta city where daytime maximum temperature was highest (Fig. 4) for all cases. This region is also a low-topography area situated tens of kilometers downwind from the central denser locations of Jakarta city. Due to the mechanical and thermal drag effects of the dense city center, sea breeze penetration is hindered thereby reducing ventilation. Moreover, with the sea breeze dominantly coming from the northeast during August months, warmer air coming from the city center tends to advected southwest from the city center (refer to the supplementary information for afternoon near-surface wind rose plots).

The spatial mean increases of 0.45 ± 0.24 and 0.93 ± 0.32 individuals under RCP26CC, and RCP85BaU, respectively. Although similar but with more intensified risk throughout the region, the future (2050s) shows varying degrees of increase spatially (Fig. 3a–c) while RCP86BaU generally shows a larger increase (suggesting twice increase than that of RCP26CC at the Southwest area of Jakarta Fig. 3a). Large increases are also obvious near the northern coasts of Jakarta (center of Fig. 3b,c). This is possibly caused by an on-going increase in residential population and urban growth within Jakarta (i.e. infilling development) implicitly considered in the study by Darmanto *et al*.^21^. Population increase entails more buildings and excess anthropogenic heat flux. With the assumed distribution of elderly population more concentrated near the coastline of Jakarta (Fig. 1b,c), this reflects higher risks to heat-related mortality in addition to already existing environmental and social problems (e.g. subsidence). From the above results, elderly citizens of Jakarta are not advised to reside near the northern coastline and southwestern areas of Jakarta in the future.

## Discussion and Conclusion

There is an increasing demand to link scientific investigations of future climate to social impacts. This work takes advantage of high-resolution climate modeling to assess heat-related health risks in cities. Utilizing modeled^21^ past and future climate to consider the effects of both urbanization and climate change in Jakarta at a kilometer-scale, heat-related mortality counts of elderly citizens were mapped in the metropolitan area.

Heat-related mortality of the elderly in Jakarta was found to increase in the 2050s because of population growth and climate change. While below 4.26~4.89% of the total elderly population remains at risk in August, a drastic increase in mortality count is expected throughout the region in August 2050s as the megacity experiences population aging and warmer climates. Mitigation and adaptation to climate change could potentially reduce heat-related elderly casualties in the future by up to 17%. Careful consideration must also be given when planning for future elderly dwellings. During August when dominant sea breeze direction is northeasterly, heat-related risks are high at the downwind southwestern area of Jakarta. It was also found that the northern coastal area of Jakarta will be a critical location in the future with larger risks estimated in those areas compared to other locations.

Uncertainties and limitations existed for the temporal or spatial change of the heat-related mortality relative risk. In this work, only one function was considered to link the daily maximum temperature to heat-related mortality relative risks. Recent investigations suggest incorporating the changes in resilience (or increasing affinity to warmer temperatures) of the elderly population with time (i.e. *RR*- *T*_*max*_ relationship is expected to time-varying). When applied to mid-latitude countries with both cold and warm seasons, previous winter conditions may influence the summer-time heat mortality^23^. “Adaptation uncertainty”^31,32^, or adaptive human behavior to climate which can possibly be greater than the uncertainty in emissions and climate modeling (SSPs and RCPs), should be considered by incorporating adaptation models in heat-mortality projections. Gosling *et al*.^31^ lists various adaptation models which may be used to estimate plausible ranges of future heat-related mortality counts.

Another limitation is that the current work utilizes the function^33^ derived specifically for Ho Chi Minh City, Vietnam. Although both Vietnam and Indonesia are neighboring countries, additional confirmation is needed regarding possible corrections to the relative risk function. Data collection and statistical analyses of heat-related mortality and morbidity rates should be conducted specifically for Jakarta, which is currently beyond the scope of this study. As the epidemiology field advances, climate-change impacts on other vulnerable population groups such as infants, young children, and underprivileged individuals will be conducted.

Finally, awareness of heat-related mortality in Southeast Asia, where is a tropical climate with typically warm season throughout the entire year, is overshadowed by more obvious environmental calamities such as flooding after typhoons, air pollution, and others. From the current findings, heat-related mortality could become more severe with urban expansion in the future. The findings of this study suggest that the high-resolution weather models with space-varying and time-varying urban parameters are needed for urban climate investigations at a global scale^34^. In this study, the methodology relies mainly on open-source modeling and commonly available global datasets (e.g. global urban expansion^35^; thus, the methodology is readily applicable for other cities.

## Methods

### Weather model

In an earlier work of Darmanto *et al*.^21^, the future climate in Jakarta was modeled considering projections of urbanization and climate change. The features of the climate model, as well as relevant settings to incorporate the scenarios are briefly discussed.

#### Model features

Simulations using the Advanced Research Weather Research and Forecasting (WRF) model^36^ ver. 3.3.1 with a modified version^37^ of the single-layer urban canopy model (UCM)^38^ were conducted. In the updated UCM module, a spatial distribution of roughness parameters, empirically derived using a new aerodynamic parameterization for urban surfaces^39^, and temporal variations in anthropogenic heating can be used as inputs. Through this module, urbanization characterized by detailed changes in the distribution of roughness parameters (zero-plane displacement height and roughness lengths for momentum and heat), sky-view factors, and anthropogenic heating were investigated. Henceforth, the aforementioned urban-representative parameters are collectively referred to in this study as “urban parameters”.

### Climate and urbanization scenarios

Future background climate and urbanization were estimated using the concept of representative concentration pathways (RCP) and shared socio-economic pathways (SSP). RCP corresponds to future climate pathways caused by four radiation forcing values by the year 2100^40^. In this study, RCP2.6 and RCP8.5 were focused with the former corresponding to very low and the latter to very high radiation forcings, respectively. Ensembles of five CMIP5 global climate models (GCMs) downloaded from the IPCC Data Distribution Centre (ipcc-data.org) were calculated for each forcing. Pseudo-Global Warming Method^41^ (PGW) was used to construct the initial and lateral boundary conditions of the climate model with the base boundary conditions taken from the NCEP FNL Operational Model Global Tropospheric Analyses^42^.

Shared-socioeconomic pathways (SSP)^43^, SSP1 and SSP3, were used to define the level of urbanization in the future. SSP1 and SSP3 were selected to represent extreme pathways for Jakarta to experience challenges in both mitigation and adaptation. To estimate the urban parameters and population dataset of 2050 (see supplementary information of this manuscript and Darmanto *et al*.^26^), a combination of historical spatial information of population, and country-level projections of GDP and total population from model outputs based on shared socio-economic pathways (SSP) were used as inputs. In accordance with the SSP narratives, the urbanization level under SSP1 assumes a “compact city” scenario with less urban sprawling compared to the SSP3 scenario where a “business-as-usual” scenario prevails. Grid-based urban growth probability was acquired using the SLEUTH urban growth model^44^. The urban growth probability was used as an additional input to estimate the future distribution of population and urban parameters^45^. A globally-available anthropogenic heat emission (AHE) dataset^46^ was used to represent additional heating per grid (1.2 km × 1.2 km) from anthropogenic activities. Using the methodology of Dong *et al*.^39^, future map of AHE were estimated using the same approach, together with the future distribution of population and present-day nighttime lights.

2-m air temperatures (or near-surface temperatures) from three case simulations were acquired from Darmanto *et al*.^21^^.^ The cases (PRESENT, RCP26CC, RCP85BaU) were selected based on their relevance to the study’s purpose and are described in Table 1. PRESENT corresponds to Jakarta’s climate and urban condition from the period 2006 to 2015. RCP26CC corresponds to a future climate with RCP2.6 emission scenario and urbanization level of “compact city” setting. RCP85BaU corresponds to a future climate with RCP8.5 emission scenario and urbanization level of “Business-as-usual” setting. Details regarding the spatial differences in urban parametric distribution among cases are available in Darmanto *et al*.^21^.

**Table 1:** Simulation cases.

Case name	Period	Emission forcing	Local urbanization
PRESENT	2006–2015	—	—
RCP26CC	2046–2055	RCP2.6	Compact city
RCP85BaU	2046–2055	RCP8.5	Business-as-usual

From the strong reliance on the aforementioned approach to SSPs, it can be said that the estimated future population, distributed urban parameters, and anthropogenic heat emissions inherently consider the influence of background climate change.

### Estimation of heat-related mortality of the elderly

#### Bias-correction of modeled temperature outputs

To precisely estimate the heat-related mortality rates, inherent biases of the modeled near-surface temperature should be corrected. This was conducted using 3-hourly observation data from three available weather stations surrounding Jakarta collected by Meteorology, Climatology, and Geophysical Agency (BMKG) of Indonesia from the August months of 2006 to 2015. The process of adjustment follows that of Piani *et al*.^47^ generally used for correcting temperature and precipitation simulated by global climate models^40^. The process relies on the calculation of a linear transfer function (TF) derived from an empirical relationship between the cumulative distribution function (CDF) of the modeled and observed temperature. The histogram of modeled temperature values for the August months was found to obey a Gaussian distribution^40^. In other words, the linear transfer function can be estimated using the percent point function, readily estimated by the corrected population mean $({\mu }_{i,j}^{\ast })$ and population standard deviation $({\sigma }_{obs}^{\ast })$ of the modeled temperature values. The bias-correction approach done for all grids of PRESENT, RCP26CC, and, RCP85BaU assumes the following equation for correcting the ${\mu }_{i,j}$ and ${\sigma }_{i,j}$ of all grids^48^.

1 $${\mu {\prime} }_{i,j}={\mu {\prime} }_{obs}+\frac{{\sigma }_{obs}}{{\sigma }_{mod}}({\mu }_{i,j}-{\mu }_{mod})$$

2 $${\sigma {\prime} }_{i,j}=\frac{{\sigma }_{i,j}{\sigma }_{obs}}{{\sigma }_{mod}}$$

where *obs* and *mod* correspond to three statistical values of temperatures individually estimated from temperatures extracted from three observation stations and its nearest grid, respectively. Indices *i* and *j* corresponds to the longitudinal and latitudinal model coordinates, respectively. All grids of each case will, thus, have three unique values of ${\mu {\prime} }_{i,j}$ and ${\sigma {\prime} }_{i,j}$ which are averaged to acquire ${\mu }_{i,j}^{\ast }$ and ${\sigma }_{i,j}^{\ast }$ for individual grids for each case. Finally, grid and case-dependent linear transfer functions $T{F}_{i,j}^{\ast }$ (represented by a linear equation comprised of a slope, value-to-be-adjusted, and an intercept) were calculated and used to acquire bias-corrected modeled hourly near-surface temperatures. $T{F}_{i,j}^{\ast }$ were estimated via the following equation,

3 $$T{F}_{i,j}^{\ast }=CD{F}^{-1}({\mu }_{i,j}^{\ast },{\sigma }_{i,j}^{\ast })$$

where the operation *CDF*^−1^ stands for the inverse of cumulative distribution function which utilizes the mean and standard deviation as inputs. $T{F}_{i,j}^{\ast }$ is in linear equation form which is comprised of the variable to be adjusted, a slope, and an intercept.

Bias-correction (Eqs. 1–3) was done for all time steps of simulated near-surface temperatures. Then, the average of daily mean and daily maximum temperature in August during the respective periods (2006–2015 for PRESENT and 2046–2055 for future cases) were calculated for all grids. Distribution of the average of daily mean and daily maximum bias-corrected near-surface temperatures (*T*_*mean*_, *T*_*max*_) are shown in Fig. 4 for PRESENT, RCP26CC, and RCP85BaU. The daily maximum bias-corrected near-surface temperatures (henceforth, *T*_*max*_) throughout the study period were later used to estimate heat-related mortality rates. See supplementary information for comparisons between the raw and bias-corrected outputs.

#### Optimum temperature and heat-related relative risk calculation

Population distribution of each case is required. Here, the assumed procedure is discussed. First, for PRESENT, population density extracted from LandScan 2013^TM^ ^49^ was resampled via nearest neighbor to match the spatial resolution of the model which is 1.2 km × 1.2 km. This process was done as well for the other cases RCP26CC and RCP85BaU. Second, a map of the elderly population (age >65) was estimated using the bulk projection of the elderly population proportion to that of Jakarta’s total population. The bulk proportion, which is broken down to age bracket, is an official estimate issued by the National Development Planning Agency of the Republic of Indonesia (Bappenas)^50^ from the year 2020 to 2035 at 5-year intervals. Regressing the data, the bulk proportion of the elderly population to the total population of each grid (*r*65) is assumed as follows,

3 $$r65=4{(10)}^{-48}{e}^{0.0525t}$$

where *t* corresponds to the target year. From analyzing statistical data provided by World Health Organization and United Nations, the crude death rate (*cdr*), which corresponds to death by all causes, specific to the elderly population was found to be equivalent to 7.09 per 100 elderly citizens per year.

*RR* was calculated as a function of the daily maximum temperature and the optimum temperature (OT). Because of the lack of *RR* function for Jakarta, the *RR* function^27^ derived for Ho Chi Minh City, Vietnam was used. The background climate and human development index of Jakarta closely resembles to that of Ho Chi Minh City in this study (see similarities in terms of climate and human development index in supplementary information). The optimum temperature was found to be 29.4 °C above which death rates were found to increase. The *RR* function which was derived for Ho Chi Minh City, Vietnam was used in this study is as follows,

4 $$R{R}_{{\rm{day}}}=\{\begin{array}{ll}1, & x < 0\\ 0.106{x}^{2}-0.0538x+1.0128, & x\ge 0\end{array}$$

where *x* = *T*_*max*day_ – 29.4. “day” corresponds to a specific day simulated. *RR*_August_ of each case was then calculated by averaging all *RR*_day_ values per grid. Finally, the total heat-related deaths for August per grid (*D*_*August*_) per case were estimated by combining Eq. 1, Eq. 2, and *RR*_August_, and the population (*pop*) of the grid (Eq. 4). To avoid overestimation, only grids with a population greater than 1000 were analyzed.

5 $${D}_{August}=Pop\times cdr\times \frac{(R{R}_{August}-1.0)}{R{R}_{August}}\times \frac{31}{365}$$

The whole process was conducted separately for all cases. Derivation of Eq. 4 is explained in the supplementary information.

Resilience change of elderly people over time was not considered in the current study. In some countries, the reduction of risk was observed^51,52^ with time. “adaptation uncertainty”^31^ must be considered in future analyses; thus, it may possibly overestimate the heat-related mortality risks.

## Supplementary information

Supplementary information.
