# Supplementary material for: Future increase in elderly heat-related mortality of a rapidly growing Asian megacity
Source: Sci Rep. 2020 Jun 9;10:9304. doi: 10.1038/s41598-020-66288-z (PMC7283254; doi:10.1038/s41598-020-66288-z)
Supplement: Supplementary file 1 — Supplementary information. [file 41598_2020_66288_MOESM1_ESM.pdf]

Future increase in elderly heat-related mortality of a rapidly growing Asian megacity

Varquez, Alvin Christopher G[a]; Darmanto, Nisrina S[a]; Honda, Yasushi[b]; Ihara, Tomohiko[c]; Kanda, Manabu[a]

[a] Department of Transdisciplinary Science and Engineering, Tokyo Institute of Technology, Japan
[b] Faculty of Health and Sport Sciences, University of Tsukuba, Japan
[c] Department of Environment Systems, University of Tokyo, Japan

Supplementary Data

1. Bias-corrected temperature verification

3-hourly observations of near-surface air temperature (2-m. above ground level) were acquired from 3 weather stations (Kemayoran Station, KMY; Tanjung Priok Station, TPR; Cengkareng station, CGK) operated by the Meteorology, Climatology, and Geophysical Agency (BMKG) of Indonesia from August 2006 to 2015. By comparing the stations' temperature data with the simulated temperatures (Darmanto et al., 2019) of its nearest computational grid, the model tends to overestimate the near-surface air temperature. As reported in Darmanto et al. (2019), the model generally overestimated the daytime near-surface temperature, with RMSE values of 1.45°C, 1.98°C, and 1.79°C for TPR, KMO, and CGK, respectively. It also resulted in a smaller bias for nighttime temperature (TPR: 0.37°C, KMO: 0.34°C, CGK: 0.41°C) than daytime temperature (TPR: 0.70°C, KMO: 1.47°C, CGK: 1.22°C).

In the current study, this bias was used to correct the temperature biases throughout the simulation domain using the approach of Piani et al. (2010) (eq. 1 and 2 of the manuscript). Here, the summary of the performance of the adjustment which mainly corrects the mean and standard deviation values is briefly discussed. The figures below summarize the performance of the adjustment.

According to Fig. 1a, the simulated values have an improved resemblance to that of the observations after bias-adjustment. This is evident from the improvement in the slope of the correlation plot. Notice as well that the bias-adjustment did not completely result in a 1:1 correlation which still suggests a possible overestimation of the simulated temperature values. Not delving into the discussion deeper, uncertainties from the observed air temperature must be acknowledged as well especially in cases where the measured temperature does not actually represent the neighborhood or model grid it belongs.

With the inherent positive temperature bias of the model and the bias-correction method used in this study, it is expected that the bias-adjusted values will be smaller than the unadjusted values (Fig. 1b; comparisons between manuscript's Fig. 4 and Fig. 1c).

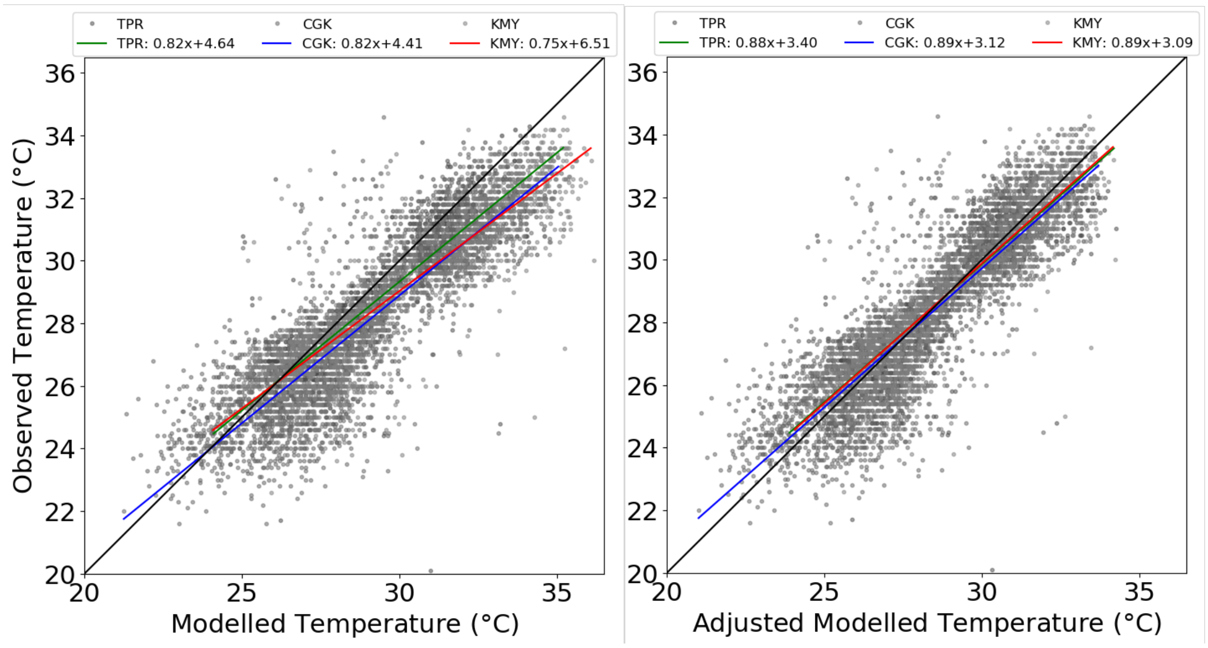

Figure 1a Comparison between simulated (nearest grid) and observed 3-hour near-surface temperatures before (Left) and after adjustment or bias-correction (Right). The colored lines correspond to a least-squares regression fitting between the horizontal and vertical values. The observation stations are denoted TPR, CGK, and KMY.

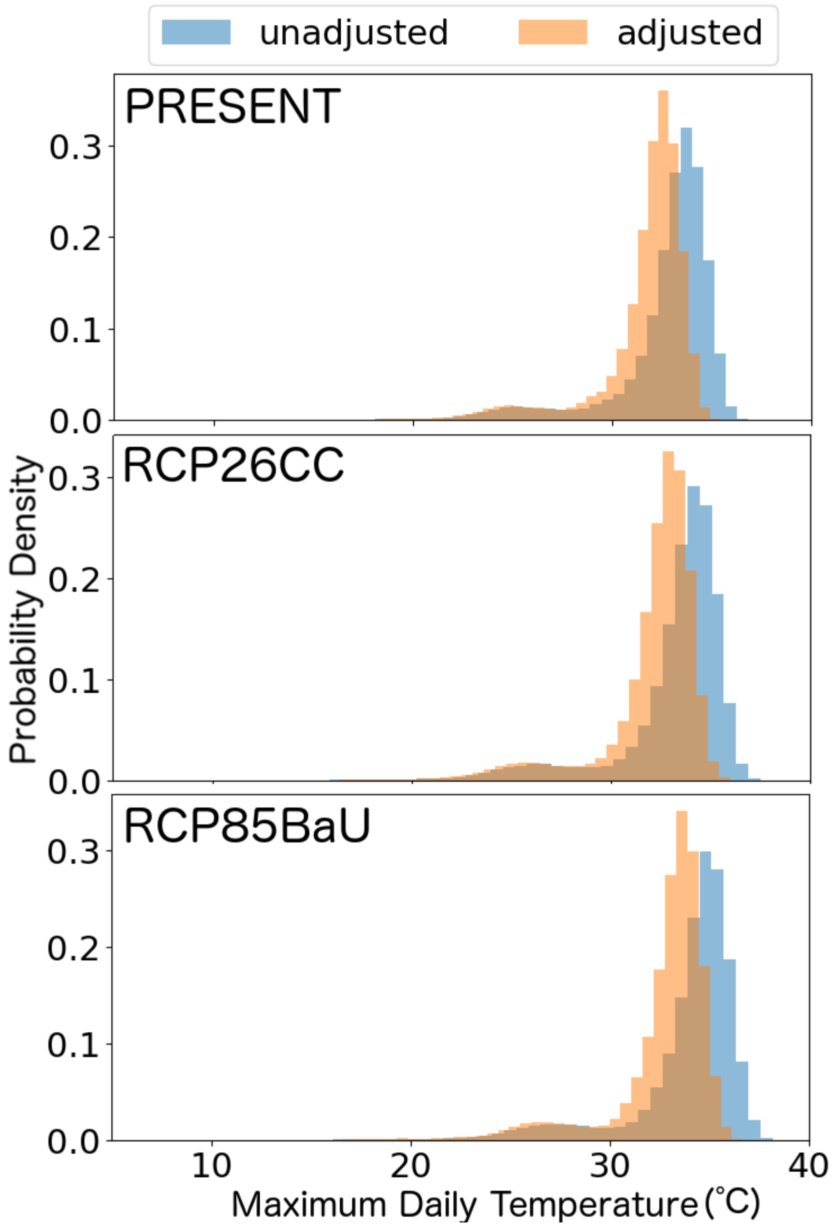

Figure 1b Histogram of maximum daily temperatures throughout the simulated domain before (unadjusted) and after (adjusted) bias-correction for the August months of 2006 (2046) to 2015 (2055). All cases shown.

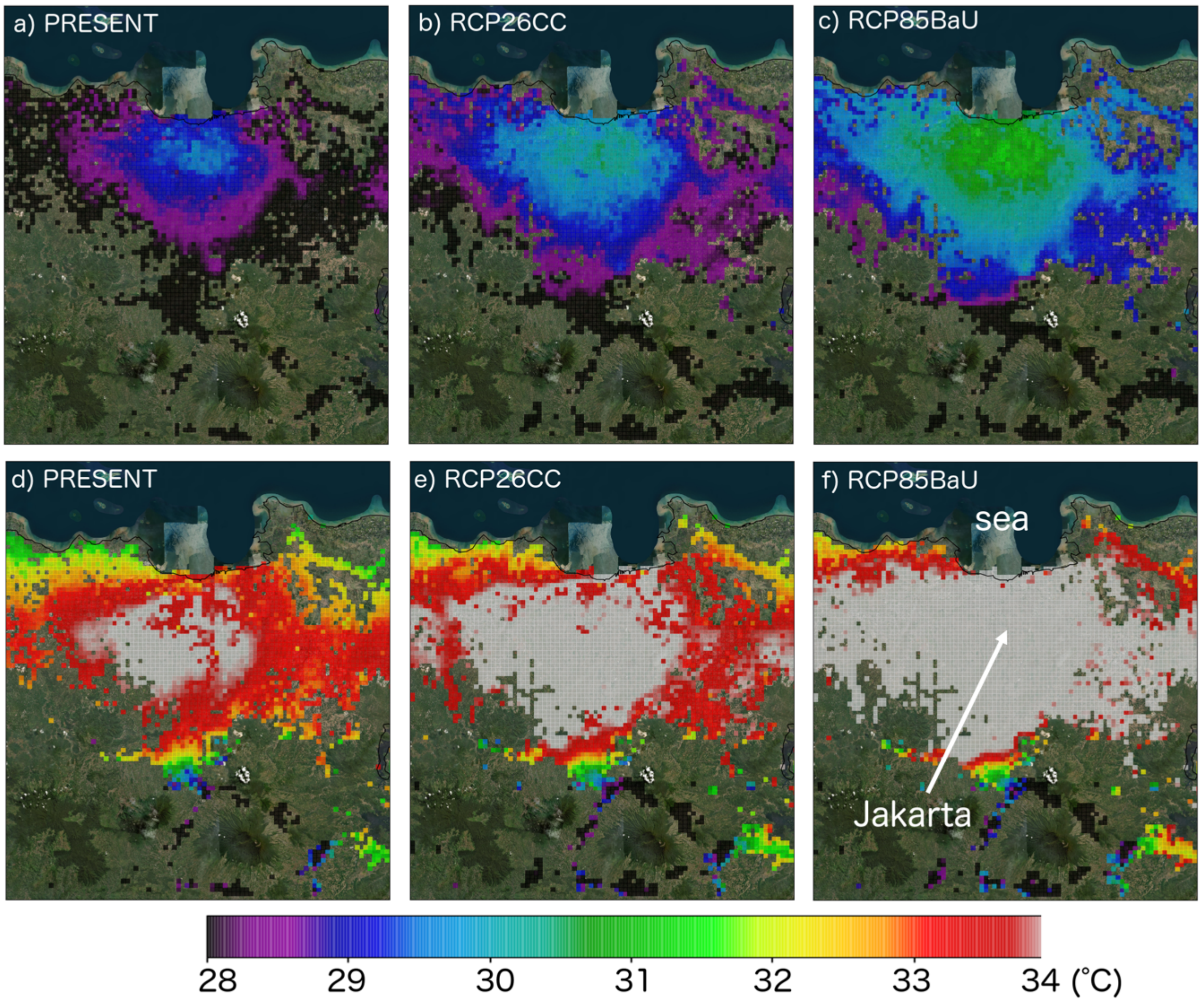

Figure 1c August-average of daily mean (a,b,c) and daily (d,e,f) maximum unadjusted near-surface temperature at urban grids of Jakarta metropolitan area for PRESENT (a,d), RCP26CC (b,e), and RCP85BaU (c,f) cases. (This figure is the same as Fig. 4 of the manuscript but using modeled temperature without bias-correction.

2. Detailed explanation and assumptions of the derivation of relative risks (RR) and heat-related mortality

In the manuscript, the derivation and definition of heat-related mortality relative risk *RR* mentioned in the manuscript are explained further in this section.

According to the textbook, "A Dictionary of Epidemiology (6th edition) by Miguel Porta, Oxford University Press", relative risk (*RR*) is defined by the ratio of two risks, usually of exposed and not exposed. Mathematically, *RR* is obtained by dividing the incidence rate (i.e. probability for a specific incident to happen) of an exposed population with the incidence rate of an unexposed population. In terms of mortality, the specific incident is crude death or deaths caused by all causes. Since the concern of this study is to estimate heat-related mortality counts or heat-related mortality *RR*, the parameter to which the population is exposed refers to ambient temperature.

The value of heat-related mortality *RR* for a certain location depends on the difference between the location's ambient temperature and a certain temperature threshold. This threshold is referred to as optimum temperature (*OT*) in the manuscript and varies with geography or

background climate. Multiple heat-related studies (stated in the manuscript) have confirmed that mortality increases when ambient temperatures exceed *OT*. *RR* is equivalent to 1.0 when the ambient temperature is equivalent to *OT* and *RR* increases depending on how much the ambient temperature exceeds *OT*. Eq. 4 in the manuscript is an example of how heat-related mortality *RR* for senior citizens (age 65 years and above) is quantified as a function of daytime maximum of ambient temperature and *OT*.

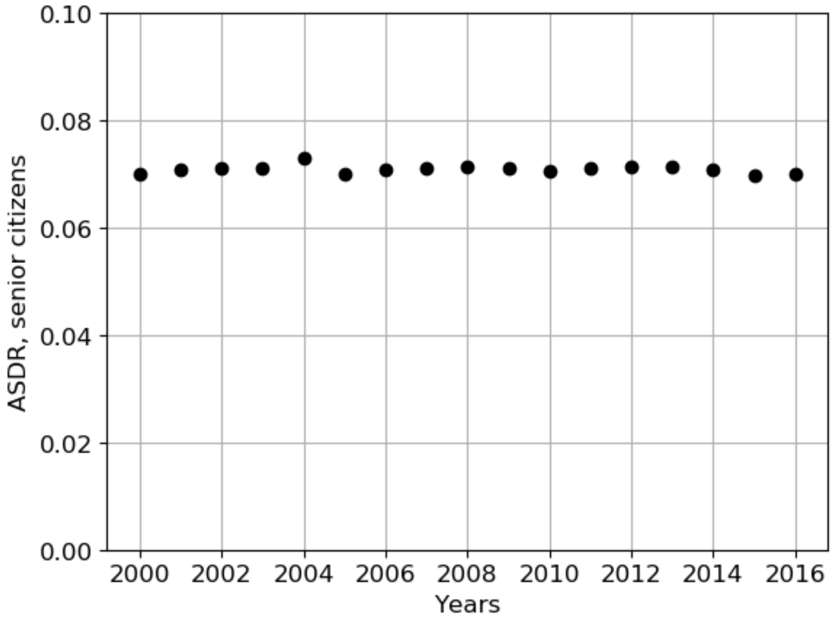

Fig. 2a Age-specific death rate (ASDR) for senior citizens age 65 years and above. Data derived from Global Health Observatory data repository maintained by the World Health Organization and World Population Prospects by the United Nations.

Table 1

Information	Source
Annual Population By Age (Tabular data)	Age Composition Data:<br>Annual Population by Age Groups – Both Sexes;<br>Annual Population by Age Groups – Male Sexes;<br>Annual Population by Age Groups – Female Sexes;<br>Link: https://population.un.org/wpp/Download/Standard/Population/<br>Accessed: January, 2020
Age-specific death rate (Tabular data)	Global Health Observatory data Repository<br>Link: https://apps.who.int/gho/data/view.main.60750<br>Accessed: January, 2020

After determining the heat-related mortality *RR* (eq. 4), the number of heat-related deaths of senior citizens is estimated. Initially, demographic databases (e.g. World Population Prospects by the United Nations, Global Health Observatory data repository by the World Health Organization) are inspected to obtain age-specific death counts in Indonesia (Table 1). Age-specific death rate (ASDR), defined as the number of deaths for a specific age group divided by the total population of the same age group, was estimated for senior citizens in Indonesia (population count with age greater or equal to 65). Fig. 2a shows a time-series of the ASDR of senior citizens estimated from the ASDR and population by age datasets for the years 2000 to 2016. The average and standard deviation for the period are 0.0709 and $\pm$ 0.000750,

respectively. Given no clear trend and significant changes in the ASDR throughout this period, the average ASDR is then assumed for the present and future cases in the study. ASDR is also the crude death rate *cdr* used in the study.

cdr, in equation form, can be expressed by the following equation while separating heat-related death counts *D* from death counts caused by other factors *Z*,

$$cdr = \frac{D+Z}{Pop} \qquad \text{eq. 2a}$$

where *D*, *Z*, and *Pop* are in terms of senior citizens' count. Meanwhile, the mathematical expression of *RR* is used as follows,

$$RR = \frac{\text{Total number of senior citizen deaths due to exposure}}{\text{Total number of senior citizen deaths without exposure}} = \frac{\frac{D+Z}{Pop}}{\frac{Z}{Pop}} = \frac{D+Z}{Z} \qquad \text{eq. 2b}$$

where exposure refers to ambient temperature values exceeding the *OT*.

Combining eq. 2a and 2b, an expression for *Z* can be derived as follows,

$$RR = \frac{D + Pop \times cdr - D}{Pop \times cdr - D} = \frac{Pop \times cdr}{Pop \times cdr - D}$$

$$Pop \times cdr - D = \frac{Pop \times cdr}{RR}$$

$$D = Pop \times cdr - \frac{Pop \times cdr}{RR}$$

$$D = Pop \times cdr \times \frac{(RR-1.0)}{RR} \qquad \text{eq. 2c}$$

Using eq. 2c, the earlier derived *cdr* and eq. 4 in the manuscript, 3.86 out of 100 senior citizens are at risk of dying when a day experiences a maximum temperature of $33°C$. Given that *Pop* and *cdr* are mid-year estimates, *D* for the August month while utilizing the mean of daily maximum temperature can be estimated by multiplying eq. 2c by the weighted proportion of the number of days of August to the year. The resulting *D* for August month will be,

$$D_{August} = Pop \times cdr \times \frac{(RR_{August}-1.0)}{RR_{August}} \times \frac{31}{365} \qquad \text{eq. 2d}$$

where D_{August} and RR_{August} corresponds to the heat-related death toll and relative risk of senior citizens (age 65 and above) for the month of August, respectively. RR_{August} can be estimated by substituting the August mean of daily maximum temperature to eq. 4 of the manuscript.

3. Wind rose plots of daily mean winds

Wind field plays a significant role in the transport of scalars within the atmosphere. The afternoon winds simulated for all August months in Jakarta tend to be north-easterly. This typical circulation is influenced by the monsoonal background of the region and sea-breeze flow. In the manuscript, winds are used to mainly attribute the relatively higher heat-risk locations at the southwest area downwind from the city center of Jakarta. Windrose plots are displayed in Fig. 3a to prove the typical northwesterly flow in the afternoon of August.

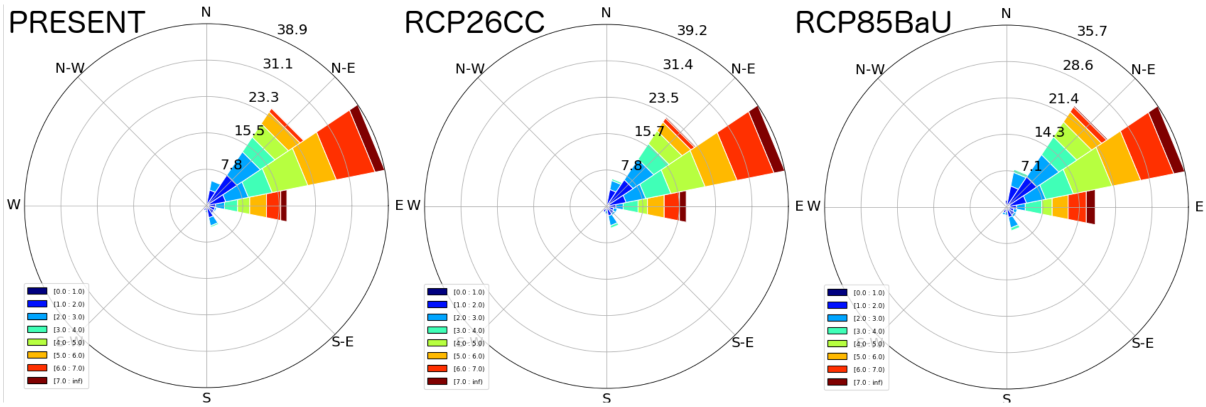

Figure 3a Windrose plots of simulated wind samples extracted from 12 PM to 6 PM throughout the analyses period under the 3 cases.

4. Climatic and human development index comparisons between Jakarta, Indonesia and Ho Chi Minh City, Vietnam

In this work, the heat-related mortality relative risks (*RR*) of Ho Chi Minh City, Vietnam was used as a proxy for the target study, Jakarta, Indonesia. The reasons for this are the lack of *RR* studies in Jakarta and the proximity between Vietnam and Indonesia in terms of geography (continental-scale), climate, and human development conditions.

Both cities are located in Southeast Asia with tropical climates, experiencing both a dry season and wet season (for Jakarta, this is called monsoonal), with no cold season. The annual mean (max) temperature for Jakarta and Ho Chi Minh City are 27.7°C (31.8°C) and 27.7°C (32.2°C), respectively. Although Ho Chi Minh City tends to have a slightly higher daytime maximum temperature than Jakarta, both cities' daytime temperature maximums between 30°C to 35°C throughout the year. For the month of August, both cities experience almost similar values of air temperature. For monthly and annual statistics of air temperature, refer to Table 4a. Given their monthly temperature variations, the continentality type of both cities are the same, hyperoceanic.

Table 4a Temperature (°C) statistics for Jakarta (J) and Ho Chi Minh City (H) (source: climatemps.com)

	City	Jul	**Aug**	Sep	Oct	Nov	Dec	Jan	Feb	Mar	Apr	May	Jun	**Yr**
Max	J	32	**32**	33	33	32	32	30	30	31	32	32	32	**31.8**
	H	31	**32**	31	31	31	31	32	33	34	35	33	32	**32.2**
Mean	J	27.5	**27.5**	28	28.5	28	28	27	27	27.5	28	28	27.5	**27.7**
	H	27.5	**28**	27.5	27.5	27	26.5	26.5	27.5	29	30	29	28	**27.8**
Min	J	23	**23**	23	24	24	24	24	24	24	24	24	23	**23.7**
	H	24	**24**	24	24	23	22	21	22	24	25	25	24	**23.5**

The human development index (HDI) is the parameter used in this study to measure the similarities of the citizens in both Jakarta and Ho Chi Minh City in terms of life expectancy, ability to acquire knowledge, and the ability to achieve a decent standard of living. From the Human Development Report Office of the United Nations Development Programme (UNDP), HDI is defined as a composite index focusing on three basic dimensions of human development: the ability to lead a long and healthy life, measured by life expectancy at birth; the ability to acquire knowledge, measured by mean years of schooling and expected years of schooling; and the ability to achieve a decent standard of living, measured by gross national

income per capita (UNDP, 2018). HDI ranges from 0.0 to 1.0, with 1.0 having the highest state of human development.

According to the 2018 statistical update of UNDP, Vietnam and Indonesia are both classified as countries with medium human development. In terms of the recent ranking, both countries share the 116th global rank with an equivalent value of 0.694 as of 2017. Comparing HDI of the past years (Table 4b) also reveal minimal differences.

Table 4b Human Development Index (HDI) for selected years in Indonesia and Vietnam (UNDP, 2018)

Country	Rank*	1990	2000	2010	2012	2014	2015	2016	2017
Indonesia	116	0..528	0.606	0.661	0.675	0.683	0..686	0.691	0.694
Vietnam	116	0.475	0.654	0.654	0.670	0.678	0.684	0.689	0.694

* Ranking by country as of 2017

Although it is ideal that a unique *RR* function is to be used for Jakarta, these functions are difficult to determine under the scope of this study. Meanwhile, an *RR* function was recently estimated for Ho Chi Minh City, which shows similarity with Jakarta in terms of climatic and human development backgrounds as shown above. Thus, for the time being until an *RR* becomes available for Jakarta under different research, the *RR* function of Ho Chi Minh City is applied for Jakarta.

Sources:

http://www.saigon.climatemps.com/, accessed on January 2020

http://www.jakarta.climatemps.com/, accessed on January 2020

Human development indices and indicators: 2018 statistical update. (United Nations Development Programme, 2018).

5. Urban parameters and population datasets used in the study

In the weather modeling of Jakarta by Darmanto et al. (2019), detailed grid-scale distributions of urban parameters including building morphological statistics and anthropogenic heat emission were used as surface boundary inputs. This was estimated for all cases; which means PRESENT, RCP26CC, and RCP85BaU have different urban parametric distributions (see Fig. 1 in Darmanto et al, 2019).

Interestingly, the preparation of their urban parametric distribution requires a detailed estimation of the population density according to each scenario, be it present, or from the shared socio-economic pathway (SSP) scenario (see Supplementary A4 of Darmanto et al, 2019). In other words, the simulation cases, PRESENT (which utilizes population density from LandScan™), RCP26CC, and RCP85BaU, were also founded upon the spatial differences in population density. Fig. 5a which was taken from the supplementary data by Darmanto et al. (2019) demonstrates the differences. For specific details on the estimation of future population distribution under RCP26CC and RCP85BaU, refer to the supplementary data by Darmanto et al. (2019).

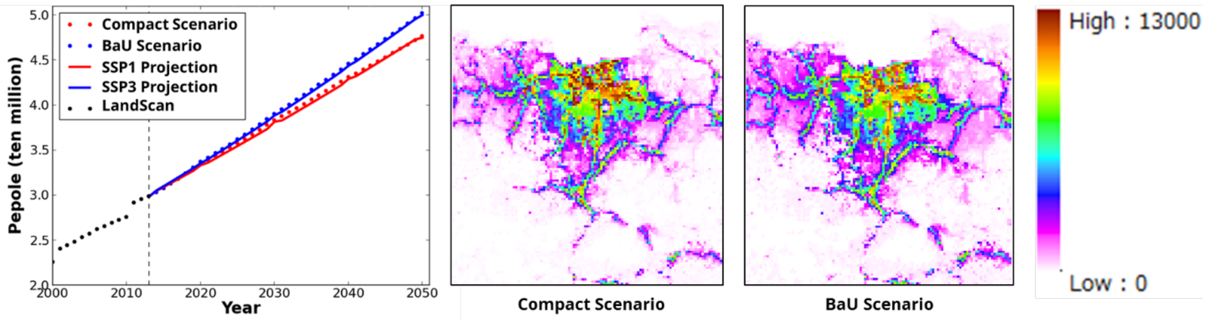

Fig. 5a population total projections (left), population density map for RCP26CC (middle) and RCP85BaU (right). Figure taken from the supplementary data of Darmanto et al. (2020) with permission.

Source:

Darmanto, N. S., Varquez, A. C. G., Kawano, N. & Kanda, M. Future urban climate projection in a tropical megacity based on global climate change and local urbanization scenarios. *Urban Climate* **29,** 100482 (2019).
